# Tuberculosis, a great masquerader: A case series unveiling rare sites of musculoskeletal involvement through imaging

**DOI:** 10.4102/sajr.v24i1.1919

**Published:** 2020-09-29

**Authors:** M. Sarthak Swarup, Shuchi Bhatt, Rajesh Rawal, Anupama Tandon, Saumya Dangwal

**Affiliations:** 1 Department of Radiology, University College of Medical Sciences and GTB hospital, Delhi, India; 2 Himalayan Institute of Medical Sciences, Swami Rama Himalayan University, Dehradun, India

**Keywords:** tuberculosis, chest wall, ultrasonography, computed tomography, sternoclavicular joint

## Abstract

Skeletal tubercular infections that do not involve the spine or large joints are rarely encountered. This case series aims to highlight the importance of imaging in diagnosing skeletal tuberculosis (TB) at uncommon sites in clinically unsuspected patients by demonstrating specific imaging findings. We present the clinical details and imaging findings of seven pathologically confirmed cases of extraspinal skeletal TB. A multimodality imaging approach including radiography, ultrasonography (USG) and computed tomography (CT) scan was used in most cases. The imaging studies revealed an infective soft tissue collection over different sites including the sternoclavicular joint, acromion process, chest wall and temporo-mandibular joint, along with destruction and erosion of the underlying or adjacent bones. In tubercular endemic countries, strong clinical suspicion should be entertained in cases presenting with a soft tissue collection, even around unusual skeletal sites.

## Introduction

Tuberculosis (TB) continues to be a major health problem in developing countries; however, the occurrence of skeletal TB is relatively uncommon. Musculoskeletal TB accounts for 1% – 3% of all the cases of TB and 15% of the extra-pulmonary variety.^1,2,3^ The involvement of the vertebra (Pott’s spine) is the most common form of skeletal TB, constituting about 50% of all cases.^1,2^ Other common sites of skeletal involvement are at the hip, knee, foot, elbow, hand and shoulder, in descending order of frequency.^1^ The sternum, ribs and the sternoclavicular joints are uncommonly affected.^3,4,5,6^ The mandible and temporo-mandibular joint appear to be the least common location affected by TB.^1^

This case series reports seven patients who presented to our department with rare sites of TB involvement such as the clavicle, sternum, acromion, chest wall and temporo-mandibular joint, most of whom had a provisional clinical diagnosis other than TB. The cases highlight the importance of imaging in establishing the diagnosis of TB, especially in clinically unsuspected cases of musculoskeletal TB. The pertinent diagnosis of musculoskeletal TB in these patients guides the extended duration of the treatment regime for these patients.

## Method

Pathologically proven cases of musculoskeletal TB were retrospectively reviewed over a 6-month period with regard to their clinical presentation, imaging findings and clinical diagnosis at the time of presentation to the radiology department. Cases of vertebral or spinal and appendicular TB were excluded and only those with an uncommon site of involvement were selected. Imaging studies pertaining to these patients included radiographs, ultrasonography (USG) and computed tomography (CT) scans. Radiographs of the chest and involved site were present for all patients. Ultrasonography had also been acquired in each case to evaluate the associated soft tissue swelling. Available non-contrast or contrast-enhanced CT scans of the chest and involved site were reviewed for evaluation of the extent of soft tissue and the underlying bone involvement. Magnetic resonance imaging (MRI) of the involved site was not performed in any of these cases, as an institutional MRI facility was not available. Ultrasonography had been used for aspiration or obtaining samples for pathological and microbiological correlation.

## Results

All the patients were immunocompetent and had no prior history of tubercular infection of any organ system. Most of the patients had been referred for a radiological work-up, with a clinical diagnosis other than TB. None had a history of close contacts of TB in the family. The chest radiographs did not reveal any discernible pulmonary lesions consistent with the diagnosis of TB. The presenting clinical symptoms and imaging findings are described for the seven patients in Table 1. All the patients were treated with multidrug anti-tubercular chemotherapy with a significant clinical response.

**TABLE 1:** Clinical presentations and imaging findings in seven cases.

Case number	Age	Sex	History and local symptoms	Clinical diagnosis	Imaging features	Final diagnosis (pathological confirmation)
1	13	Female	Swelling and discharging ulcer over the right sternoclavicular joint, following penetrating trauma	Post-traumatic infection	USG: A hypo-echoic collection with internal echoes and some hyper-echoic foci, reaching up to the skin surface (Figure 1a). CT: Revealed involvement of the medial end of the clavicle along with an adjacent hypodense collection and necrotic mediastinal lymphadenopathy (Figure 1b and 1c). The sternal aspect was spared.	Sternoclavicular TB (Fine needle aspirate from the lesion revealed caseating granulomas with epithelioid cells, which are specific for Tuberculosis [Figure 1d]. Examination of the sample from the collection was found to be positive for AFB. The patient showed a good clinical response to anti-tubercular chemotherapy)
2	23	Female	Swelling in the right parotid region with trismus	Parotid abscess	USG: A hypo-echoic collection in the right parotid region was present outside the parotid gland in the masseter muscle. CT: Confirmed the collection (3.8 x 2.7 cm) in the right masseter and showed a bony lesion in the right mandibular condyle. There was partial lysis of the bone with cortical erosion. The adjacent collection extended into the right temporo-mandibular joint (Figure 2a, 2b and 2c).	Temporo- mandibular joint TB (Aspiration of pus proved to be AFB positive; subsequent culture was confirmatory for *M. tuberculosis*)
3	32	Male	Progressive swelling over the right shoulder region with no pain	Soft tissue tumour	CT: Destruction of acromion process, unsuspected on plain radiography, with an adjacent bulky subscapularis muscle (Figure 3a and 3b). A collection was revealed on subsequent ultrasonographic examination (Figure 3c).	Acromial TB with TB lymphadenitis (Fine needle aspirate from associated enlarged axillary lymph nodes revealed epithelioid cell granulomas with caseous necrosis (Figure 3d). A sample from the collection was also examined, which was positive for AFB)
4	15	Female	Cough for 1 month, evening rise of temperature with subtle swelling over the chest	Pulmonary TB	USG: A hypo-echoic collection in the upper anterior chest wall in relation to the left sternoclavicular joint (Figure 4a). CT: Acquired for evaluation of the lung, demonstrated tree in bud nodular opacities in the superior segment of the right lower lobe (Figure 4b) and necrotic mediastinal nodes, which suggested the possibility of TB. The involvement of both sternoclavicular joints was also evident (Figure 4c and 4d).	Pulmonary TB and bilateral sternoclavicular joint TB (Sputum was positive for AFB) (Although staining of smears to identify AFB is non-specific, it is still considered as a reference standard for the diagnosis of TB in endemic areas. The patient was commenced on anti-tubercular chemotherapy with a dramatic response).
5	23	Female	Swelling over the right chest wall with low-grade fever and fatigue.	Empyema necessitans	Contrast-enhanced CT chest was conducted for the clinical suspicion of pulmonary TB complicated by empyema. Computed tomography revealed a soft tissue collection in the right chest wall with a normal underlying rib and pleural cavity (Figure 5a and 5b). Healed fibronodular lesions were seen in both lung apices. The dorsal spine did not show any tubercular lesion, nor were there any clinical signs and symptoms consistent with Pott’s spine.	Chest wall soft tissue TB (Tubercular nature of the collection was confirmed on aspiration and subsequent CB-NAAT)
6	14	Female	Pain and swelling in the left breast	Breast lesion could not be characterised further	USG: The breast tissue appeared normal. A retromammary collection was present with no obvious extension deeper into the chest wall. CT: Revealed a large chest wall abscess involving the left pectoralis muscle with the involvement of the 5th rib and its costal cartilage (Figure 6). A similar but smaller collection was seen in the subscapularis muscle.	Chest wall TB (Pus aspirated was AFB positive; Tubercular aetiology was proved by growth of *M. tuberculosis* in the culture specimen)
7.	18	Male	Swelling over the left side of the chest wall with a recent history of trauma	Chest wall haematoma	CT: confirmed the chest wall collection on the left side with underlying subtle rib erosion (Figure 7a and 7b).	Chest wall TB (Aspirated collection was positive for TB on CB-NAAT)

USG, Ultrasonography; CT, computed tomography; TB, tuberculosis; AFB, Acid-fast bacilli; CB-NAAT, Cartridge based nucleic acid amplification test.

**FIGURE 1:**
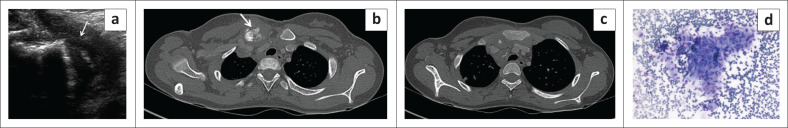
A 13-year-old female with swelling over the right sternoclavicular joint. Ultrasonography (a) of the affected area shows a hypo-echoic collection (white arrow) with internal echoes, reaching up to the skin surface. Post-contrast axial computed tomography bone window images (b, c) revealed the involvement of the medial end of the clavicle (white arrow) and adjacent hypodense collection with sparing of the sternal aspect. Fine needle aspirate (d) shows a well-formed caseating granuloma composed of the epithelioid cells with characteristic slipper-shaped nuclei and indistinct cell boundaries (May-Grünwald-Giemsa x 200).

**FIGURE 2:**
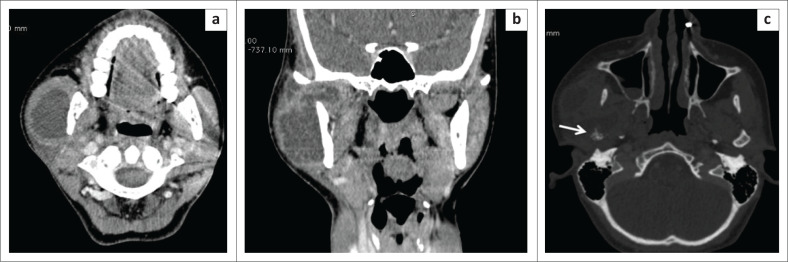
A 23-year-old female with swelling in the right parotid region. Post-contrast axial (a) and coronal (b) computed tomography images demonstrate a hypodense collection in the right masseter extending into the temporo-mandibular joint. Axial computed tomography bone window image (c) shows the involvement of the right mandibular condyle with partial lysis of the bone and cortical erosion (white arrow).

**FIGURE 3:**
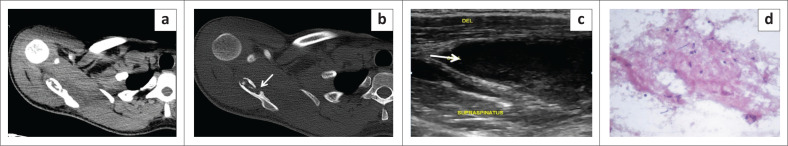
A 32-year-old male with swelling over the right shoulder region. Axial computed tomography images (a, b) show bony destruction of the right acromion process (white arrow) with an adjacent bulky subscapularis muscle. Subsequent ultrasound image (c) shows a hypo-echoic collection in between subscapularis and deltoid muscles. Fine needle aspirate (d) from an associated enlarged axillary lymph node shows caseous necrosis seen as acellular debris against a pauci-cellular background (Papanicolaou stain x 400).

**FIGURE 4:**
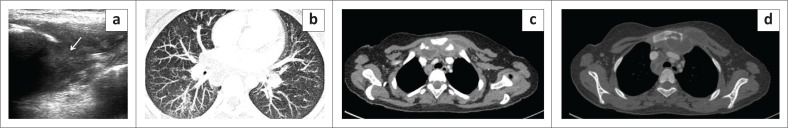
A 15-year-old female with a subtle swelling on the chest wall. Ultrasonography image (a) shows a hypo-echoic collection (arrow) in the upper anterior chest wall in relation to the left sternoclavicular joint. Axial lung window computed tomography image (b) shows tree in bud nodular opacities in the right lower lobe superior segment. Axial soft tissue (c) and bone (d) window computed tomography images show necrotic mediastinal nodes and involvement of both sternoclavicular joints with hypodense collections and adjacent bony erosions.

**FIGURE 5:**
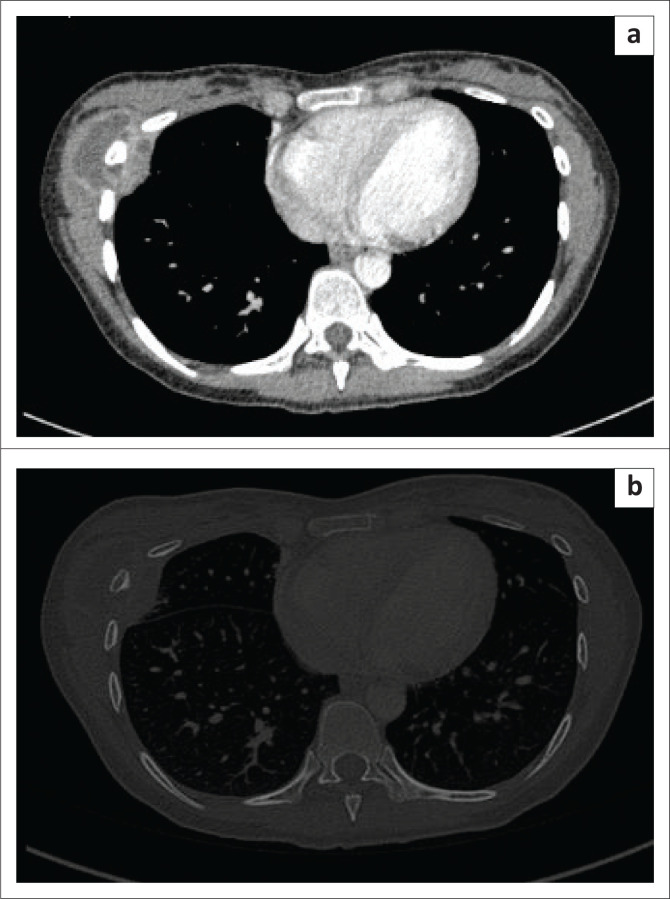
A 23-year-old female with swelling over the right chest wall. Post-contrast computed tomography axial soft tissue and bone window images (a, b) show a peripherally enhancing, hypodense soft tissue collection in the right chest wall with no obvious involvement of the underlying ribs or pleural cavity.

**FIGURE 6:**
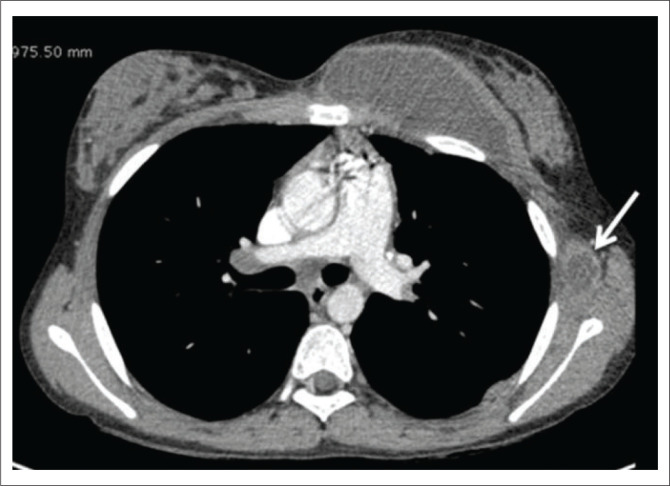
A 14-year-old female presenting with a left breast swelling. Axial post-contrast computed tomography image shows a large chest wall abscess involving the left pectoralis muscle with involvement of the costal cartilage and 5th rib. A similar but smaller collection was seen in the left subscapularis muscle (white arrow).

**FIGURE 7:**
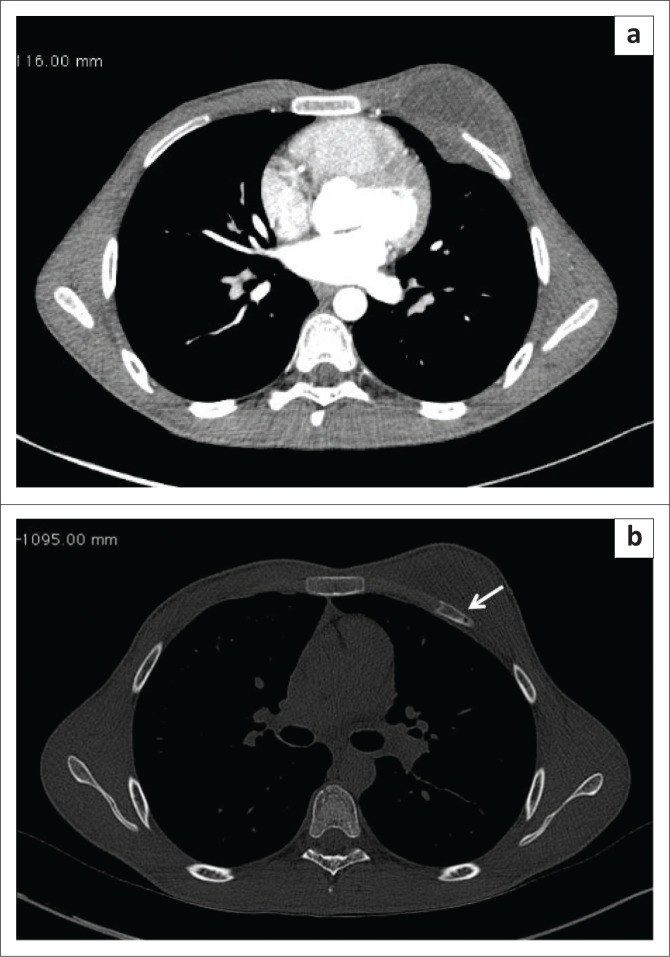
An 18-year-old male with swelling on the left chest wall. Post-contrast axial computed tomography soft tissue and bone window images (a, b) illustrate the chest wall collection on the left side with underlying subtle rib erosion (white arrow).

## Discussion

Although TB remains a major health hazard in the developing world, it is not uncommonly encountered in developed countries in recent times. The increase in the prevalence of TB worldwide, especially the involvement of unusual sites, is because of immigration from endemic areas, increased prevalence of immunosuppression (Human immune deficiency virus [HIV] infection and use of immunosuppressive drugs), and the emergence of multidrug and extensively drug-resistant strains of *Mycobacterium tuberculosis*. However, none of the patients were found to be HIV positive in this series. The other speculations about the increase in such cases might be because of the easy accessibility to healthcare facilities, increased use of cross-sectional imaging modalities and confirmation of the diagnosis by better diagnostic laboratory services.

Although the manifestations of TB are usually limited to the chest, the disease can affect virtually any organ system in the body. Musculoskeletal TB constitutes a relatively uncommon group of TB. The common sites of skeletal involvement are the spine, hip joint, knee joint, foot, elbow, hand and shoulder joint.^1^ The affection of the chest wall with the involvement of sternum, ribs and the sternoclavicular joints are uncommonly encountered.^3,4,5,6^ Tuberculosis of the chest wall constitutes 1% to 5% of all cases of musculoskeletal TB.^7^ Sternoclavicular joint TB accounts for only 1% – 2% of all cases of peripheral tubercular arthritis.^4^ Tuberculosis of the ribs constitutes 2% of the total cases of musculoskeletal TB.^6^ Tuberculous osteomyelitis of the sternum and scapula are exceedingly rare.^8,9^ Tubercular arthritis of the temporo-mandibular joints are very rare; only a few cases have been reported.^10^

Multimodality imaging with USG, multidetector computed tomography (MDCT) and MRI play an important role in the diagnosis of TB, as lesions may be subtle in the initial stage and, therefore, mostly not appreciated on radiographs. However, imaging alone is insufficient in reaching a conclusive diagnosis and pathological/microbiological analysis of the tissue sample is required for making a definitive diagnosis. The cartridge-based nucleic acid amplification test (CB-NAAT/GeneXpert) is an automated molecular technique, which not only detects *M. tuberculosis*, but also rifampicin resistance. It has been increasingly used for bacteriological confirmation from different tissue samples both in pulmonary and specific forms of extra-pulmonary TB. This is particularly useful in cases with extra-pulmonary involvement and paucibacillary disease.^11^

The unusual locations depicted in this case series are sternum, sternoclavicular joint, ribs, acromion and mandible. Positive radiographic signs occur much later than the presenting clinical features. Abscesses or sinuses are usually present before the focus can be detected radiologically. Computed tomography demonstrates abnormalities earlier than plain radiography and therefore detects the bony changes, which may be missed on radiography. Computed tomography scan is especially helpful in evaluating the extent of osseous and joint involvement, degree of bone destruction and demonstration of periosteal reaction or sequestrum.^12^ Besides this, it can also demonstrate the extension of the infection into the surrounding soft tissue, presence and extent of associated soft tissue abscesses and calcifications within the cold abscess.^12^

The CT features of sternoclavicular TB include bony destruction, soft tissue masses crossing fascial planes with rim enhancement representing an abscess or diffuse enhancement representing granulation tissue and the presence of calcifications.^3,4^ The condition usually starts from the medial extremity of the clavicle.^1^ Underlying pleuro-parenchymal tubercular involvement is also commonly seen. Similar findings are demonstrated on CT in cases with acromio-clavicular joint involvement. The disease may start from the lateral extremity of the clavicle or from the tip of the acromion.^1^

Tubercular involvement of ribs presents as either bony erosions with disruption of the cortical margin or frank destruction, with adjacent abscess formation.^6,7^ There may be focal expansion of the involved rib with or without periosteal reaction. Tuberculous abscesses of the chest wall can involve the sternum, costochondral junctions, rib shafts, costovertebral joints and the vertebrae.^7^ They are most frequently found at the margins of the sternum and along the rib shafts. A tuberculous retromammary abscess appears as a focal, smoothly marginated, inhomogeneous, hypodense lesion with a surrounding enhancing rim.^13^ A direct fistulous communication with the pleura or a destroyed rib fragment found in a tuberculous abscess can be helpful in differentiating it from other types of retromammary abscess.^13,14^ In the case of chest wall TB, the detection of underlying pleuro-parenchymal disease is often helpful in suggesting the diagnosis.^3^

Computed tomography demonstration of tubercular involvement of osseous and soft tissue in these rare sites has important management implications as this will determine the total duration of anti-tubercular treatment in a patient with pulmonary TB. Patients with only pulmonary TB are usually treated with 6 months of multidrug chemotherapy, whereas patients with osteoarticular TB are treated for a minimum period of 9 to 12 months. It may extend up to 18 to 24 months depending upon the site of skeletal involvement, clinico radiological response and national/local management guidelines.^12^ Therefore, a careful scrutiny of the bones on CT is warranted. In patients without any clinical suspicion of pulmonary TB, detecting typical pulmonary lesions of TB (active or old healed) acts as supportive evidence of tubercular involvement of bone and soft tissue of the chest wall.

Our patients demonstrated similar imaging findings, better documented on CT as lytic destruction or erosion of the sternum, clavicle, acromion, ribs and mandibular condyle. Soft tissue collections were detected around the bony involvement and this was also demonstrated on USG, where accessible. Ultrasonography showed the abscess as a hypo-echoic collection with posterior acoustic enhancement and varying degrees of internal heterogeneity. Some authors have also highlighted the use of USG as a cost-effective and useful modality to assess rib destruction/irregularity and associated soft tissue abscess in chest wall TB.^6^ In addition, USG provides real-time guidance for obtaining a tissue sample for pathological confirmation. The need for image guidance in accurately obtaining the tissue specimen from deep-seated collections/pathology, which is not appreciated clinically, is an important aspect of management, as the histopathological demonstration of caseous necrosis in the granulomas and microbiological identification in culture provides definite evidence of TB.^2^

Musculoskeletal TB demonstrates a variety of clinical and radiologic features and can mimic several other disease entities.^15^ Common conditions in the differential diagnosis are low-grade pyogenic infection, rheumatoid disease, myeloma or secondary metastatic deposits. Most of our patients were suspected to have a clinical diagnosis other than TB, reiterating the fact that TB is the great masquerader. Imaging was required to unveil the diagnosis or to confirm the clinical diagnosis. Only two patients (cases 4 and 5) had a clinical suspicion of pulmonary TB and CT scans were performed primarily for the evaluation of pulmonary involvement, which also revealed the associated bony and soft tissue involvement of chest wall. Computed tomography plays a principal role in the detection of an unsuspected lesion or in demonstrating the true nature of the lesion. We would like to highlight the importance of MDCT, which could change the clinical diagnosis (as in cases 1, 2, 3, 6 and 7), and detect the associated rib or bony lesion thereby changing the duration of anti-tubercular treatment instituted to the patients (as in cases 4 and 5). Hence, in resource-limited settings where MRI is unavailable, as in our facility, the imaging modality of greatest importance in detecting musculoskeletal TB at unusual locations is CT as it can show the extent of the bony involvement in great detail, as well as the associated soft tissue collections. Although USG can be a useful modality in evaluating the soft tissue affection especially in accessible sites, it is limited in its ability to demonstrate the underlying bony involvement.

Magnetic resonance imaging (with gadolinium enhancement) is the modality of choice for early detection of tubercular arthritis and osteomyelitis, even if its early findings are non-specific.^12^ Magnetic resonance imaging can reveal intraosseous involvement earlier than the other imaging modalities by demonstrating marrow changes in osteomyelitis. Magnetic resonance imaging can easily identify soft-tissue masses, differentiate between granulation tissue and an abscess and assess the degree of marrow involvement and cortical erosion. However, bone anatomy and the extent of bony destruction, calcifications and sequestra are better appreciated at CT scan.^2,12^

Although the majority of earlier investigators have highlighted the role of CT scan in various unusual sites of tubercular osteomyelitis and arthritis, few workers have also described the MRI abnormalities in musculoskeletal TB. Magnetic resonance imaging shows the marrow changes in the sternum, clavicle and other bones as a hypo-intense area on T1-weighted and hyper-intense area on T2-weighted sequences, along with an associated enhancing soft tissue abscesses. In cases of arthritis, MRI can also demonstrate joint effusion, abnormalities within the articular cartilage and subchondral bone. Although MRI has the advantage of non-exposure to ionising radiation, as well as excellent delineation of soft tissue and marrow pathology, clinicians in some parts of the developing world, similar to our facility, still prefer a CT study because of accessibility and affordability issues. Computed tomography can raise the suspicion in clinically unsuspected cases or support the diagnosis in clinically suspected patients, thereby guiding the clinical management in most cases.

## Conclusion

A non-specific, often indolent clinical presentation along with a low index of suspicion may result in a delayed diagnosis of extraspinal musculoskeletal TB. Therefore, the possibility of TB should be considered even if the site of involvement is atypical such as at the sternoclavicular joint, clavicle, acromion, chest wall or temporo-mandibular joint, particularly in endemic regions or in the immigrant population from endemic areas. Undertaking appropriate imaging helps the radiologist to raise the suspicion of tubercular aetiology when a non-specific soft tissue collection is discovered adjacent to bones and joints, even in unusual locations.

Imaging has important management implications by providing guidance to obtain a tissue sample and determining the total duration of anti-tubercular treatment by demonstrating associated skeletal involvement in cases of pulmonary TB. Computed tomography can be an immensely useful modality in resource-constrained environments where MRI is unavailable. Ultrasound remains an easy, cost-effective initial imaging option.

The final diagnosis is usually established by pathological and/or microbiological tests. Tuberculosis should be considered even in the presence of atypical radiologic findings in high-risk populations in an appropriate clinical setting. Typical radiologic manifestations help to diagnose musculoskeletal TB in unusual locations and in clinically unsuspected cases.
